# Silencing of SPARC represses heterotopic ossification via inhibition of the MAPK signaling pathway

**DOI:** 10.1042/BSR20191805

**Published:** 2019-11-12

**Authors:** Qianjun Wang, Qianqian Yang, Ali Zhang, Zhiqiang Kang, Yingsheng Wang, Zhentao Zhang

**Affiliations:** Department of Orthopedics, The 89th Hospital of Chinese People’s Liberation Army, Weifang 261021, P.R. China

**Keywords:** ALP activity, Heterotopic ossification, Mineralized nodules, Mitogen-activated protein kinase signaling pathway, OCN content, Secreted protein acidic and rich in cysteine

## Abstract

Heterotopic ossification (HO), the pathologic formation of extraskeletal bone, can be disabling and lethal. However, the underlying molecular mechanisms were largely unknown. The present study aimed to clarify the involvement of secreted protein acidic and rich in cysteine (SPARC) and the underlying mechanism in rat model of HO. The mechanistic investigation on roles of SPARC in HO was examined through gain- and loss-of-function approaches of SPARC, with alkaline-phosphatase (ALP) activity, mineralized nodules, and osteocalcin (OCN) content measured. To further confirm the regulatory role of SPARC, levels of mitogen-activated protein kinase (MAPK) signaling pathways-related proteins (extracellular signal-regulated kinase (ERK), c-jun N-terminal kinase (JNK), p38, nuclear factor κ-B (NF-κB), and IkB kinase β (IKKβ)) were determined. Bone marrow mesenchymal stem cells were treated with pathway inhibitor to investigate the relationship among SPARC, MAPK signaling pathway, and HO. The results suggested that SPARC expression was up-regulated in Achilles tendon tissues of HO rats. Silencing of SPARC could decrease phosphorylation of ERK, JNK, p38, NF-κB, and IKKβ. Additionally, silencing of SPARC or inhibition of MAPK signaling pathway could reduce the ALP activity, the number of mineralized nodules, and OCN content, thus impeding HO. To sum up, our study identifies the inhibitory role of SPARC gene silencing in HO via the MAPK signaling pathway, suggesting SPARC presents a potential target for HO therapy.

## Introduction

Heterotopic ossification (HO) represents a process by which extraskeletal bone forms in soft tissues [1]. HO is considered a complication following trauma and genetic disease, and it can give rise to not only disability, but also mortality [2]. Some risk factors have been identified to be associated with HO after primary total hip arthroplasty, including male sex, lateral approach, and total cemented implants [3]. The outbreak of HO has the features of stiffness, swelling, pain, and warmth, affecting the patient’s quality of life while also presenting a bad effect on the patients owing to its influence on movement [4]. Also, it has been revealed that HO is a disorder characterized with little known pathogenesis, insufficient effective prevention, and treatment approaches, also accompanied by a high rate of disability [5]. Therefore, it is necessary to have a better understanding of the molecular mechanisms underlying HO to identify novel detection biomarkers, thereby improving the current treatment strategies.

The secreted protein acidic and rich in cysteine (SPARC), also referred to as Osteonectin, appears to have a significant effect on abilities of formation, maturation, and survival of osteoblast [6]. SPARC, commonly known as an indicator of bone formation, also has the ability to promote calcium deposition as well as the differentiation and survival of osteoblasts [7]. Also, SPARC represents an osteogenic marker gene, and RANKL could promote Pi-induced cardiac fibroblast calcification *in vitro* by activating SPARC [8]. A previous study has since demonstrated that SPARC is highly positive in ligament fibers and the periosteal membrane attached to the apex of the styloid process [9]. Besides, it has also been documented that SPARC has the potential to increase the extent of p38 mitogen-activated protein kinase (MAPK) and MAPK-activated protein kinase 2 (MAPK-APPK2) phosphorylation and can activate the p38 MAPK-heat shock protein 27 (HSP27) signaling pathway [10]. MAPK signaling pathway, activated by trauma-induced HO serum, has the ability to mediate the osteogenic differentiation of human adipose-derived stromal/stem cells [11]. More importantly, Activin A receptor type I (ACVR1) can increase the nuclear factor κ-B (NF-κB) and p38MAPK activity, thus creating a proinflammatory state, while NF-κB/MAPK activation is the basis of ACVR1-mediated inflammation in human HO [12]. ACVR1, belonging to type I bone morphogenetic protein receptor family, has been revealed to be abnormally activated in a mouse model of fibro-dysplasia ossificans progression, potentially leading to HO. Besides, ACVR1 knockout has been reported to induce osteogenic differentiation. However, the effect SPARC has on HO and its underlying mechanism remains inadequately understood, owing to a lack of evidence supporting this hypothesis. Therefore, the aim of the present study was to thoroughly investigate the regulatory effects associated with SPARC on HO, along with the underlying mechanism connected with the MAPK signaling pathway.

## Materials and methods

### Establishment of HO rat model

Thirty healthy male Wistar rats were used in the present study (weight: 200–250 g; Changsha Tianqin Biotechnology Co., Ltd., Changsha, China). Rats were anesthetized with an intraperitoneal injection of 30 μg/g pentobarbital under aseptic conditions. The left crus posterolateral approach was used. The rats were then randomly divided into the sham group (the skin was cut and stitched after exposing the Achilles tendon), and the HO group (vascular clamp was used to repeatedly clamp five times on both sides of fractured Achilles tendon to cause trauma, and left Achilles was unstitched, and the skin incision was stitched) (15 rats in each group). All rats were raised under the same conditions and fed normally [5].

### Hematoxylin and Eosin staining

Following 7 weeks of modeling, all rats were killed by carbon dioxide asphyxiation. Achilles tendon of each group was subsequently fixed using the fixative solution for 3 min, placed in a 15% sucrose solution for 30 min, and added with 30% sucrose solution in 4°C refrigerator overnight. Next, Achilles tendon was added with 30% sucrose solution for 2 h, embedded the mixture in an optimal cutting temperature (OCT), frozen, sliced, and dried overnight at room temperature. Afterward, the sections were placed in a 37°C oven for 30 min and then placed at room temperature for 30 min. Sections were then stained using Hematoxylin for 50 s, washed in hydrochloric acid–ethanol solution for three to six times, placed in Eosin solution for 20 s, and treated with 95% ethanol for 1 s, 100% ethanol twice for 1 s, xylene twice for 3 s, and finally sealed with neutral gum.

### Immunohistochemistry

Paraffin sections were later dewaxed, dehydrated with gradient alcohol, repaired with the antigen repair solution, and added with the normal goat serum blocking solution (C-0005, Shanghai Haoran Biotechnology Co., Ltd., Shanghai, China) at room temperature for 20 min. The sections were then incubated with primary rabbit polyclonal antibody to SPARC (ab14174, 1:1000, Abcam Inc., Cambridge, MA, U.S.A.) overnight at 4°C. Next, the sections were cultured with goat anti-rabbit Immunoglobulin G (IgG) (ab6785, 1:1000, Abcam Inc., Cambridge, MA, U.S.A.) secondary antibody for incubation at 37°C for 20 min. Subsequently, the sections were coupled with horseradish peroxidase (HRP)-labeled streptavidin protein working solution (0343-10000U, Beijing Yimo Biotechnology Co., Ltd., Beijing, China) for additional incubation under similar parameters. The routine procedures were performed according to a previous literature [13]. Microscopically, the cytoplasm showing yellow stain was viewed as positive expression of SPARC. Five high magnification views for each section were randomly selected with 100 cells per view for observation: SPARC-positive cells <10% was negative, the SPARC-positive cells were ≥10% and <50% was positive, the number of SPARC-positive cells >50% for strong positive.

### Isolation and culture of bone marrow mesenchymal stem cells

Bone marrow (5 ml) was extracted from the anterior superior iliac spine of multiple myeloma patients (from the 89th Hospital of Chinese People’s Liberation Army) under aseptic conditions. Bone marrow and heparin anticoagulation (1:1) were placed on the Ficoll separation and centrifuged at 2200 rpm for 20 min. Next, the mononuclear cell suspension in the intermediate junction was aspirated, resuspended with Dulbecco’s Modified Eagle’s Medium (DMEM) and centrifuged at 1000 rpm for 10 min, resuspended with calcium- and magnesium-free phosphate buffer saline (PBS), and centrifuged again at 1000 rpm for 10 min. Cells were then inoculated in a 25-ml culture bottle at a concentration of 4 × 10^8^ l^−1^, and inoculated in 0.05 volume fraction of CO_2_ incubator at 37°C with saturated humidity. The culture medium was DMEM-low glucose (LG) containing 10% newborn calf serum, 100 U/ml penicillin, and 100 mg/l streptomycin. Next, cells were passaged at a ratio of 1:2 or 1:3.

### Immunophenotyping of bone marrow mesenchymal stem cells

Direct immunofluorescence (DIF) and flow cytometry analysis (FCM) were used. Monoclonal antibodies against CD29 (BD, 557332), CD106 (BD, 561679), CD34 (BD, 550761), CD105 (BD, 560839), and CD45 (BD, 560975) were selected. Bone marrow mesenchymal stem cells (BMSCs) were detached with concentration adjusted to 10^10^ l^−1^. A total of 100 μl cells was added with monoclonal antibody vascular endothelial growth factor (VEGF) at room temperature for 30 min and tested. Phycoerythrin or fluoro-isothiocyanate (FITC) labeled isotype IgG served as negative control (NC).

### Cell grouping

Multiple myeloma MSCs were transfected with sh-SPARC plasmid, oe-SPARC plasmid or relative oe-NC plasmid, or added with 1 μmol/l p38 MAPK inhibitor SB203580, 1 μmol/l extracellular signal-regulated kinase (ERK) MAPK inhibitor PD98059 (S1177200mg, Selleck, U.S.A.), 1 μmol/l c-jun N-terminal kinase (JNK) MAPK inhibitor SP600125 (S1460200mg, Selleck, U.S.A.) or 1 μmol/l dimethyl sulfoxide (DMSO) as a control individually or together. sh-RNA vector piU6 and overexpression vector pcDNA3.1 were purchased from AddGene and synthesized by Nanjing GenScript Biotechnology Co., Ltd. (Nanjing, China).

### RNA isolation and quantification

The total RNA was extracted using miRNeasy mini Kit RNA (Beijing China Ocean Co., Ltd., Beijing, China) (spin-column method) and then reverse transcribed into cDNA by using PrimeScript reverse transcription (RT) Kit (RR036A, Takara Biotechnology Co., Ltd., Dalian, China). SPARC mRNA primers were designed and synthesized by Invitrogen, NY, CA, U.S.A. (Table 1). The primers were added into ABI7300 real-time fluorescence quantitative polymerase chain reaction (qPCR) (7300, Applied Biosystems (ABI), Foster City, CA, U.S.A.) for amplification according to the instructions of SYBR® Premix Ex Taq™ II kit (RR820A, Takara Biotechnology Ltd., Dalian, China). Glyceraldehyde-3-phosphate dehydrogenase (GAPDH) was used as an internal reference and the relative transcription level of the target gene was calculated by 2^−ΔΔ*C*^_t_ method [14].

**Table 1:** Primer sequences for RT-qPCR

Gene	Primer sequences
*SPARC*	Forward: 5′-GCACTGGGCTACAGGAAAGT-3′
	Reverse: 5′-TCCGACCATTCCTTCCGTTG-3′
*GAPDH*	Forward: 5′-GAACATCATCCCTGCATCCA-3′
	Reverse: 5′-CCAGTGAGCTTCCCGTTCA-3′

All the above gene sequences were from NCBI. Abbreviation: RT-qPCR, reverse transcription qPCR.

### Western blot analysis

The total protein was then extracted using a radio immunoprecipitation assay (RIPA) Kit (R0010, Beijing Solarbio Life Sciences Co., Ltd, Beijing, China), separated by polyacrylamide gel electrophoresis followed by its transfer on to a nitrocellulose membrane by wet transfer method. Next, the membrane was blocked using a 5% bovine serum albumin (BSA), added in with diluted primary rabbit antibodies: SPARC (1:500, ab55847), p38 (1:1000, ab32142), ERK1/2 (1:1000, ab115799), JNK (1:2000, ab179461), phosphorylated-P38 (p-P38) (1:1000, ab47363), phosphorylated-ERK (p-ERK) (1:1000, ab76299), phosphorylated-JNK (p-JNK) (1:5000, ab131499), NF-κB (1:2000, ab16502), phosphorylated-NF-κB (p-NF-κB) (1:1000, ab28856), IkB kinase β (IKKβ) (1:1000, ab124957), and phosphorylated-IKKβ (p-IKKβ) (1:1000, ab59195) (all from Abcam Inc., Cambridge, MA, U.S.A.) for incubation for a total of 4 h overnight. The membrane was then incubated, this time with the HRP-labeled rabbit anti-human IgG (F020218, Beijing Biolab Technology Co., Ltd., Beijing, China). The membrane was reacted with an enhanced chemiluminescence reagent (ECL808-25, Biomiga San Diego, CA, U.S.A.) for 1 min. X-ray (36209ES01, Shanghai qcbio Science & Technologies Co., Ltd., Shanghai, China) was conducted to observe the results. GAPDH (1:1000, ab181602, Abcam Inc., Cambridge, MA, U.S.A.) was used as an internal reference and the ratio of gray value of the target band to the internal reference band was used as the relative expression of the protein.

### Osteogenic differentiation

BMSCs were inoculated into the six-well plate, with each well being added in with 3 ml of low-sugar DMEM containing 12–15% fetal bovine serum, 100 U/ml penicillin, and streptomycin. In addition, 0.25 g/l vitamin C, 1 μm dexamethasone, and 10 mM β-phosphoglycerate was added to facilitate osteogenic differentiation. The bone marrow solution was mixed thoroughly and then inoculated into the six-well plate (2–3 ml per well) in an incubator at 37°C with 5% CO_2_. Following a culture for 48 h, the supernatant was discarded, and half medium was changed. The whole medium was changed after 96 h. The medium was changed every 2–3 days with the unattached cells gradually discarded. Then, cells were passaged at a ratio of 1:3 and labeled as P1, and the aforementioned procedure was repeated to obtain P2 and P3 cells.

### Alkaline-phosphatase staining

Sterile slides were placed in the cell culture dishes, and cell suspension was added during passage for preparation of cell slides. When the cells had covered the slides, they would be fixed using a cold propyl alcohol for 10 min, followed by incubation at 37°C for up to 4–6 h, immersed in 2% cobalt nitrate for 3–5 min, fixed in 1% ammonium sulfide for 2 min, naturally dried, and sealed. Go-mori cobalt-modified method was applied to measure the activity of Alkaline-phosphatase (ALP), with the positive sites transforming from brown to black. The ALP staining results of the cells were observed and the ALP activities were measured using the Image-Pro Plus software (Media Cybernetics, Silver Spring, MD, U.S.A.).

### Alizarin Red S staining

BMSCs were washed twice with 1× PBS (pH = 7.2, without calcium and magnesium), fixed with 70% ethanol for 1 h, rinsed with distilled water three times, and incubated with 40 mmol/l Alizarin Red S at 37°C for 60 min. Following three washes with distilled water, the number and volume of mineralized nodules were observed under a microscope and then quantified.

### Radioimmunoassay

The supernatant of the passaged cells was then collected on the 6th, 12th, and 18th days following the simvastatin treatment. The content of osteocalcin (OCN) in the supernatant was detected using the OCN radioimmunoassay kit (XFFM079D, Shanghai Xinfan Biotechnology Co., Ltd., Shanghai, China). The counts per minute (cpm) were measured using a counter. The content of OCN in the sample was reflected by the standard curve.

### Statistical analysis

All statistical analyses were performed using SPSS 21.0 software (IBM Corp., Armonk, NY, U.S.A.). Measurement data were expressed using the mean ± standard deviation. Comparison between two groups was analyzed by *t* test and corrected by Welch. The Shapiro–Wilk method was used to test the normal distribution of data among multiple groups, and measurement data consistent with the normal distribution were analyzed using one-way analysis of variance (ANOVA). Fisher’s Least Significant Difference (LSD) test was applied for pairwise comparison. Comparisons of data concerning skewed distribution were analyzed using a non-parametric Kruskal–Wallis test. *P*<0.05 was considered statistically significant.

## Results

### Characterization of HO rat model

**Figure 1:**
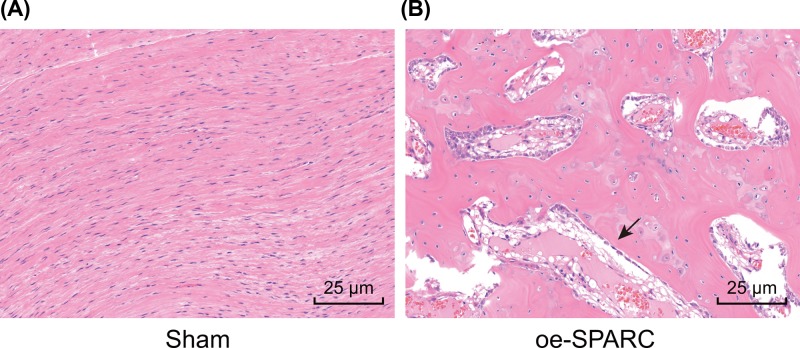
HE staining of Achilles tendon tissues HE staining of Achilles tendon tissues (400×) in the sham group (**A**) and the oe-SPARC group (**B**) indicating that the rat model is established successfully. The arrow referred to chondrocytes, bone lacunae and trabecular bone structures in the HO group.

On the first day after modeling, two rats in the HO group died. The remaining rats were in good condition, no incision infection was observed, and incision healed well. The Hematoxylin and Eosin (HE) staining was conducted in both sham and HO groups following remodeling for 7 weeks. According to the results of the HE staining (Figure 1A,B), there was no cartilage and bone formation detected in the Achilles tendon of the sham group; chondrocytes, bone lacunae, and trabecular bone structures were observed in the HO group, suggesting that the HO rat model was successfully established, with the success rate of rat modeling being 80%.

### SPARC expresses at a high level in HO tissues

**Figure 2:**
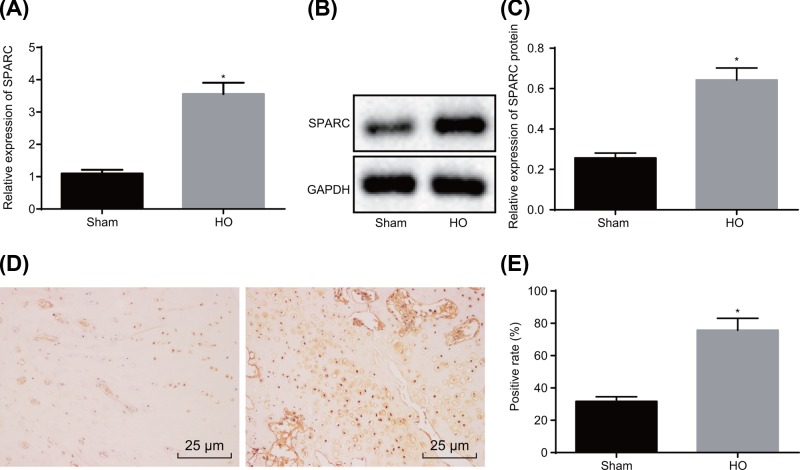
SPARC is up-regulated in Achilles tendon tissues of HO rats (**A**) The mRNA expression of SPARC in Achilles tendon of rats in each group were determined by RT-qPCR. (**B,C**) The protein expression of SPARC normalized to GAPDH in Achilles tendon of rats in each group were determined by Western blot analysis. (**D,E**) The SPARC-positive rate was detected by immunohistochemistry, *n*=10 (400×). *P<0.05 *vs.* the sham group. Data above were measurement data, which were expressed as mean ± standard deviation. Comparisons of two groups were assessed by unpaired *t* test. The experiment was repeated three times independently.

Subsequently, the expression of SPARC was measured in HO tissues by RT-qPCR and Western blot analysis. When compared with the sham group, the SPARC expression was significantly enhanced in the HO group (*P*<0.05) (Figure 2A–C). The SPARC-positive rate was further detected by immunohistochemistry. The results (Figure 2D,E) showed that the SPARC-positive rate in the HO group (75.83%) was significantly higher than the expression detected in the sham group (31.67%) (*P*<0.05). The aforementioned results suggested that SPARC was highly expressed in HO tissues.

### Multiple myeloma MSCs are successfully isolated and cultured

**Figure 3:**
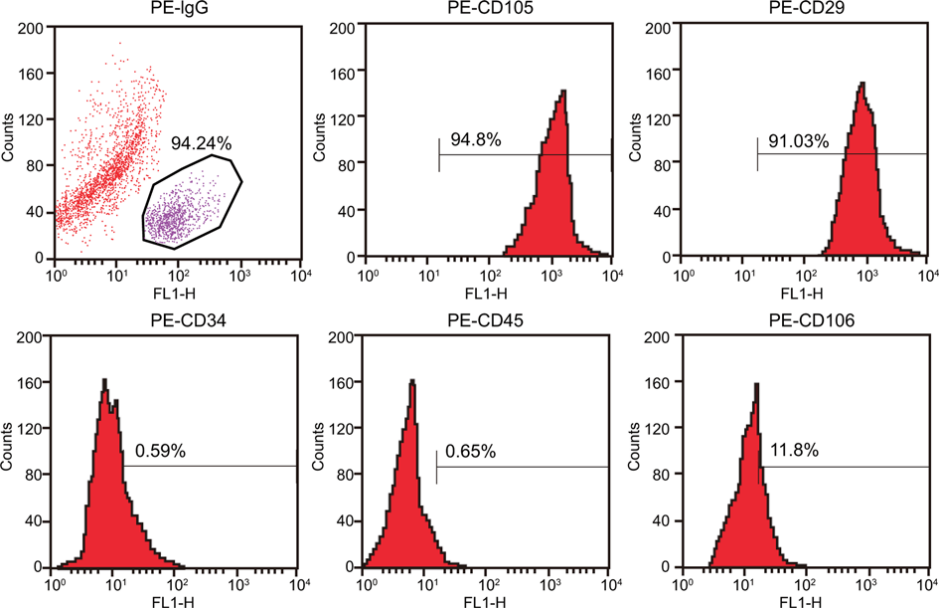
MSCs of multiple myeloma are successfully isolated and cultured

The immunophenotype of BMSCs was characterized. As shown in Figure 3, the second generation of MSCs of multiple myeloma showed no expression of surface markers of hematopoietic cells, such as CD34 and CD45, and displayed low expression of CD106, high expression of CD29, and the surface markers of MSCs (CD105). Collectively, these characteristics helped reveal that MSCs of multiple myeloma were successfully isolated and cultured.

### Silencing of SPARC impedes HO progression

**Figure 4:**
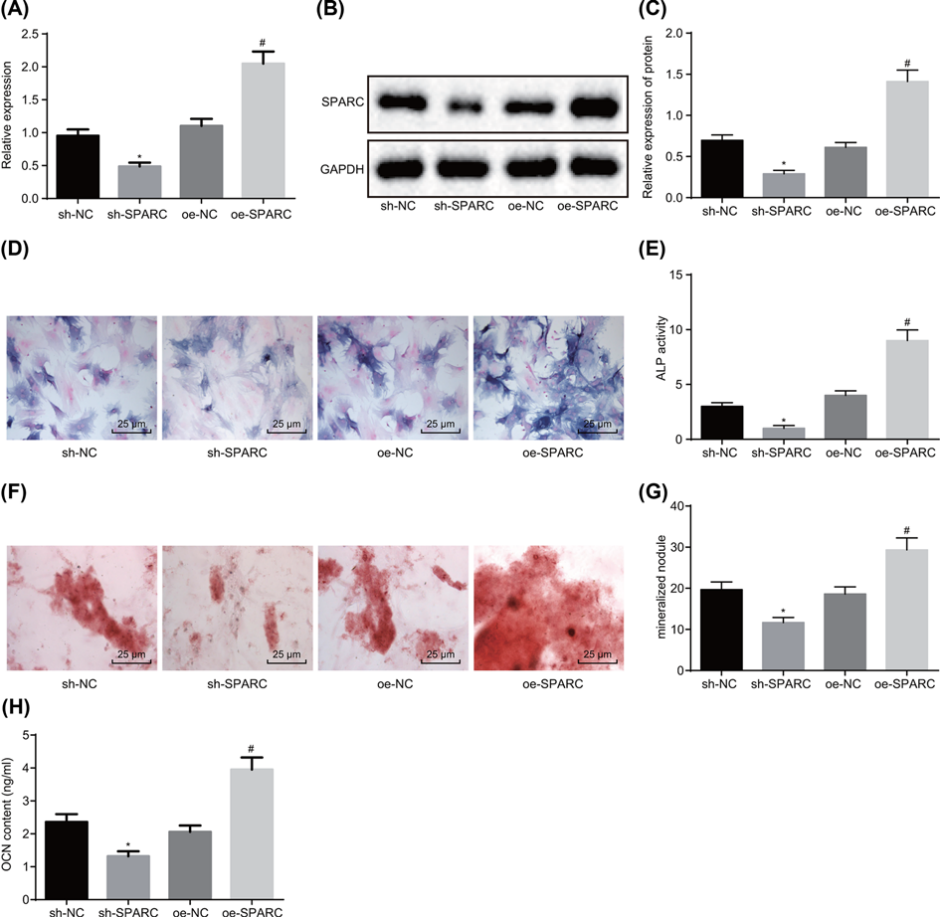
Silencing of SPARC could hinder progression of HO (**A**) RT-qPCR was carried out to measure the mRNA expression of SPARC of cells in each group. (**B,C**) Western blot analysis was performed to determine the protein expression of SPARC of cells in each group normalized to GAPDH. (**D,E**) ALP staining was conducted to detect the ALP activity in each group (400×). (**F,G**) Alizarin Red S staining was used to detect the number of mineralized nodules (400×). (**H**) The OCN content of each group. *P<0.05 *vs.* the sh-NC group (BMSCs treated with sh-NC), ^#^*P*<0.05 *vs.* the oe-NC group (BMSCs treated with oe-NC). Data in the figure were measurement data, which were expressed as mean ± standard deviation. Comparison among multiple groups was analyzed by one-way ANOVA. The experiment was repeated three times independently.

Following the treatment of BMSCs with oe-SPARC and sh-SPARC, osteogenic differentiation was induced, with the expression of SPARC later detected by RT-qPCR and Western blot analysis. The results (Figure 4A–C) showed that in contrast with the cells treated with sh-NC, the expression of SPARC was significantly decreased in the cells treated with sh-SPARC. The cells treated with oe-SPARC exhibited higher expression of SPARC than those treated with oe-NC. Next, ALP staining, Alizarin Red S staining, and radioimmunoassay were conducted to detect the osteogenesis. It was shown that (Figure 4D–H) the ALP activity, the number of mineralized nodules, and OCN content significantly diminished in cells treated with sh-SPARC (*P*<0.05), while it was reciprocal in cells treated with oe-SPARC (*P*<0.05). These results showed that silencing SPARC exerted suppressive effects on HO.

### SPARC activates the MAPK signaling pathway

**Figure 5:**
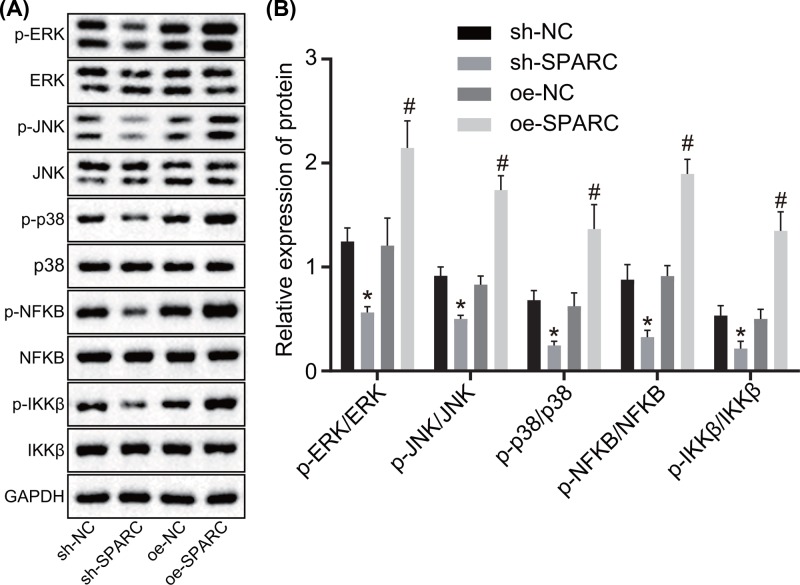
SPARC promotes the activation of the MAPK signaling pathway (**A,B**) Western blot analysis was carried out to determine the expression of MAPK-related proteins normalized to GAPDH. *P<0.05 *vs.* the sh-NC group (BMSCs treated with sh-NC), ^#^*P*<0.05 *vs.* the oe-NC group (BMSCs treated with oe-NC). Data in the figure were measurement data, which were expressed as mean ± standard deviation. Comparison among multiple groups was analyzed by one-way ANOVA. The experiment was repeated three times independently.

It has been well documented that SPARC could activate the MAPK signaling pathway [10]. Afterward, Western blot analysis was performed to determine the protein expression of MAPK-related factors following transfection with oe-SPARC and sh-SPARC. It was subsequently revealed that (Figure 5A,B) in contrast with the cells treated with sh-NC, the extent of ERK, JNK, p38, NF-κB, and IKKβ phosphorylation remarkably decreased in the cells treated with sh-SPARC; in contrast with the cells treated with oe-NC, the extent of ERK, JNK, p38, NF-κB, and IKKβ phosphorylation was enhanced in the cells treated with oe-SPARC. These results demonstrated that SPARC was able to activate the MAPK signaling pathway.

### Inhibition of the MAPK signaling pathway exerts therapeutic effect on HO

**Figure 6:**
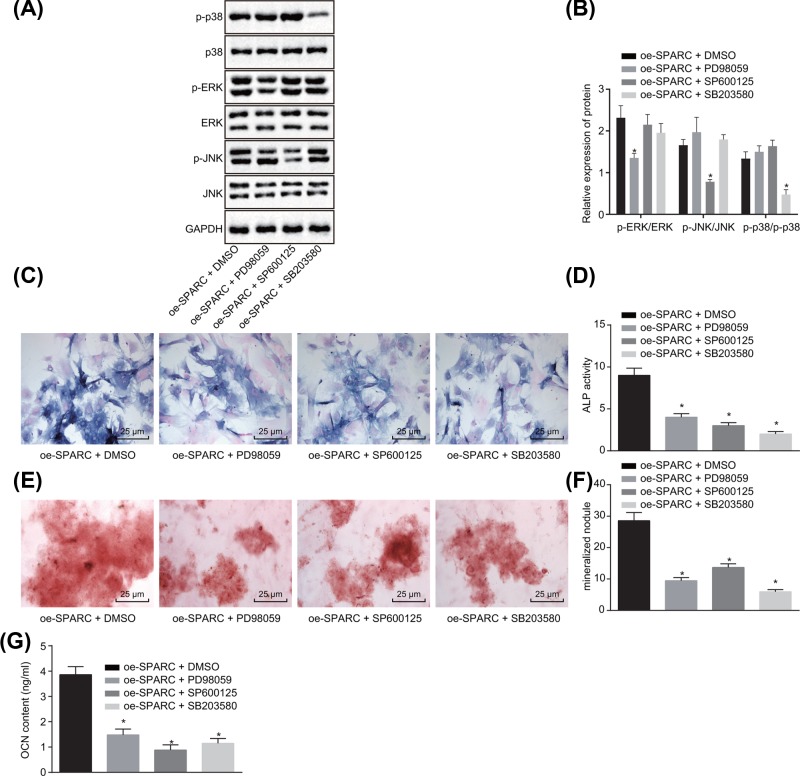
Inhibited MAPK signaling pathway exerts therapeutic effect on HO (**A,B**) Western blot analysis was carried out to determine the expression of MAPK-related proteins of BMSCs in each group normalized to GAPDH. (**C,D**) ALP staining was conducted to detect the ALP activity in each group (400×). (**E,F**) Alizarin Red S staining was used to detect the number of mineralized nodules (400×). (**G**) The OCN content of each group. *P<0.05 *vs.* the oe-SPARC + DMSO group (BMSCs treated with oe-SPARC + DMSO). Data in the figure were measurement data, which were expressed as mean ± standard deviation. Comparison among multiple groups was analyzed by one-way ANOVA. The experiment was repeated three times independently.

Subsequently, the present study further investigated the relationship among SPARC, MAPK signaling pathway, and HO. Western blot analysis (Figure 6A,B) was used to detect the expression changes in MAPK-related proteins. In comparison with the cells treated with oe-SPARC + DMSO, the cells treated with oe-SPARC and ERK MAPK inhibitor PD98059 showed reduced ERK phosphorylation, while the extent of JNK and p38 phosphorylation had no significant differences. Besides, the extent of JNK phosphorylation was diminished but the extent of ERK and p38 phosphorylation showed no significant differences in the cells treated with oe-SPARC and JNK MAPK inhibitor SP600125. Furthermore, the extent of p38 phosphorylation was notably diminished but the extent of ERK and JNK phosphorylation had no significant difference in the cells treated with oe-SPARC and p38 MAPK inhibitor SB203580.

Next, ALP staining, Alizarin Red S staining, and radioimmunoassay (Figure 6C–G) were conducted to detect the osteogenesis. Relative to the cells treated with oe-SPARC + DMSO, the ALP activity, the number of mineralized nodules as well as OCN content of ERK were significantly diminished (*P*<0.05), but there were no significant differences detected in JNK and p38 in the cells treated with oe-SPARC and PD98059. Additionally, the ALP activity, the number of mineralized nodules, and OCN content of JNK were diminished (*P*<0.05), but there was no significant difference in ERK and p38 in the cells treated with oe-SPARC and SP600125. Moreover, the ALP activity, the number of mineralized nodules, and OCN content of p38 were significantly diminished (*P*<0.05), but there was no significant difference in ERK and JNK in the cells treated with oe-SPARC and SB203580. The results suggested that suppression of the MAPK signaling pathway exerted a therapeutic effect on HO.

## Discussion

HO has been reported difficult to diagnose at an early stage due to the lack of effective signs and symptoms, as well as there being limited treatment methods for HO [1,15]. Therefore, there is an urgent need to explore the oncogenic mechanisms underlying HO development. It has been noted that several genes exert functions on HO, for example, decreased GNAS can lead to HO via the activation of Hedgehog signaling [2]. GNAS, a transcriptionally complex locus that can encode Gsα, has been implicated in progressive osseous heteroplasia, a rare developmental disorder of HO [16]. This study explored the potential roles of the SPARC and the MAPK signaling pathway in HO, which supports the notion that the silencing SPARC could inhibit the activation of the MAPK signaling pathway and effectively inhibit the development of HO.

SPARC was highly expressed in HO tissues, and silencing SPARC inhibited the MAPK signaling pathway as evidenced by the decreased levels of ERK, JNK, p38, NF-κB, and IKKβ, as well as the extent of ERK, JNK, p38, NF-κB, and IKKβ phosphorylation. In the process of bone formation, SPARC can be secreted by osteoblasts, with an elevation in SPARC expression exhibited in osseous tissues [17]. SPARC is a matricellular protein that has been expressed at a high level in bone cells and it is essential in bone remodeling to promote osteoblast differentiation [18]. Additionally, SPARC is highly expressed in osteoblasts in both normal bone and HO tissues in comparison with preosteoclasts and young osteocytes [19]. A previous study has shown that a conditioned medium of SPARC treated bone marrow stromal cell can activate p38-MAPK signaling in prostate cancer [20]. It is manifested that there are three subgroups of MAPKs including ERK, JNK, and p38 [21]. IKKβ, an inhibitor of NF-κB, has been recognized to participate in various developmental and physiological procedures in vertebrates as well as the mediation of spindle bipolarity, cellular transformation, and chromosomal stability [22,23]. SPARC has been demonstrated to increase p38 MAPK phosphorylation and activate the p38 MAPK/HSP27 signaling pathway, thus promoting glioma cell migration on fibronectin [24]. Consistent with our study, it has also been proven that SPARC can elevate the extent of p38 MAPK, MAPKAPK2, and Ser^78^-HSP27 phosphorylation [10]. The aforementioned evidence suggested that SPARC may be involved in HO and silencing SPARC could inhibit the activation of the MAPK signaling pathway.

Furthermore, the present study showed that silencing SPARC could exert a suppressive effect on HO via the inhibition of the MAPK signaling pathway, which was supported by a decrease in ALP activity, mineralized nodules, and OCN content. ALP activity and OCN content were the early and late markers in the process of osteogenic differentiation, respectively [25]. A previous study revealed that SPARC plays a key role in the process of osteogenic differentiation of the initial crystal growth on stem cells [26]. Also, SPARC is an indicator of bone formation that has the potential to promote calcium deposition as well as differentiation and survival of osteoblast [7]. Moreover, it has been revealed that SPARC possesses the ability to promote osteoblast growth and proliferation, while also regulating matrix mineralization in healing wounds and bone formation [27]. SPARC-related modular calcium binding 1 has been revealed to be closely involved in osteogenic differentiation of BMSCs and has the potential to promote bone regeneration *in vivo* [28]. It has been suggested that the MAPK/ERK signaling pathways may be related to the Nell-1 induced osteogenic differentiation of pre-osteoblasts on titanium surfaces [29]. ERK1/2, p38 MAPK, and JNK osteogenic signaling pathways have been demonstrated to participate in mechanical stress-induced osteoblastic differentiation [30]. The phosphatidylinositol 3-kinase/Akt/mammalian target of rapamycin (mTOR) signaling pathway has been identified to play important roles in various cellular procedures [31]. The mediation of NF-κB has been indicated to depend on Akt and managed by mTOR and Raptor in relation to IKK [32]. Runx1 has been elucidated to be involved in the mediation of the NF-κB signaling pathway by cross-talking with IKK in the cytoplasm [33]. The disruption of Runx1 has been reported to induce bone marrow failure due to transcriptional dysregulation and DNA repair defect [34]. Moreover, functional deficiency of mutant Runx2 has been confirmed to suppress amelogenesis and osteogenesis [35]. The existing literature has noted that activation of NF-κB can activate activator protein 1 to promote the activation of the MAPK signaling pathway [36]. Another study has revealed that mineral trioxide aggregate-treated bone marrow stromal cells can increase ALP activity, enhance the mineralization, and up-regulate the expression of odonto/osteoblastic markers via the activation of the MAPK signaling pathway [37], which is consistent with the current study. From the evidence above, we can subsequently conclude that silencing SPARC can repress HO by suppressing the MAPK signaling pathway.

**Figure 7:**
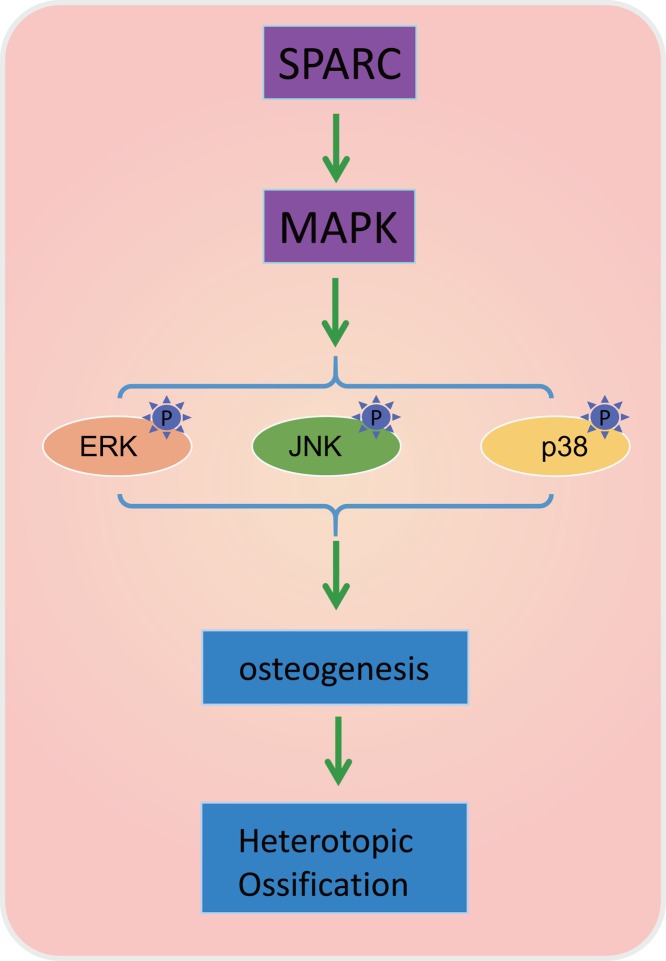
The regulatory mechanism of SPARC in HO SPARC promotes the activation of the MAPK signaling pathway to increase the phosphorylation of ERK, JNK, p38, NF-κB and IKKβ, thereby accelerating the occurrence of HO in soft tissues and playing a role in the treatment of HO.

In conclusion, our study demonstrated that silencing of SPARC could suppress the HO development through the inhibition of the MAPK signaling pathway (Figure 7). This provides a new insight into the role and molecular mechanism of SPARC involved in HO. Besides, this study has helped to advance our understanding of the pathophysiology of HO formation, as well as prophylaxis and treatment strategies. Nevertheless, due to the lack of report on the potential role of SPARC in HO progression, further studies of this mechanism are required.

## Abbreviations

ABI: Applied Biosystems
ACVR1: Activin A receptor type I
ALP: alkaline-phosphatase
BMSC: bone marrow mesenchymal stem cell
DMEM: Dulbecco’s Modified Eagle’s Medium
DMSO: dimethyl sulfoxide
ERK: extracellular signal-regulated kinase
GAPDH: glyceraldehyde-3-phosphate dehydrogenase
HE: Hematoxylin and Eosin
HO: heterotopic ossification
HRP: horseradish peroxidase
HSP27: heat shock protein 27
IgG: immunoglobulin G
IKKβ: IkB kinase β
JNK: c-Jun N-terminal kinase
MAPK: mitogen-activated protein kinase
mTOR: mammalian target of rapamycin
NC: negative control
NF-κB: nuclear factor κ-B
OCN: osteocalcin
PBS: phosphate buffer saline
qPCR: quantitative polymerase chain reaction
RT: reverse transcription
SPARC: secreted protein acidic and rich in cysteine

## References

[1] Agarwal S. et al. (2016) Inhibition of Hif1alpha prevents both trauma-induced and genetic heterotopic ossification. Proc. Natl. Acad. Sci. U.S.A. 113, E338–E347 10.1073/pnas.1515397113 26721400 PMC4725488

[2] Regard J.B. et al. (2013) Activation of Hedgehog signaling by loss of GNAS causes heterotopic ossification. Nat. Med. 19, 1505–1512 10.1038/nm.3314 24076664 PMC3917515

[3] Pavlou G. et al. (2012) Risk factors for heterotopic ossification in primary total hip arthroplasty. Hip. Int. 22, 50–55 10.5301/HIP.2012.9057 22362505

[4] Convente M.R. et al. (2018) Depletion of mast cells and macrophages impairs heterotopic ossification in an Acvr1(R206H) mouse model of fibrodysplasia ossificans progressiva. J. Bone Miner. Res. 33, 269–282 10.1002/jbmr.3304 28986986 PMC7737844

[5] Xu J.C. et al. (2017) Establishment of heterotopic ossification via sharp instrument injury in rats. J. Musculoskelet. Neuronal Interact. 17, 456–460 28250250 PMC5383774

[6] Zhang L. et al. (2013) The rhPTH treatment elevates plasma secreted protein acidic and rich in cysteine levels in patients with osteoporosis. Osteoporos. Int. 24, 1107–1112 10.1007/s00198-012-1956-3 22419369

[7] Huang R.S., Brown R.E. and Buryanek J. (2014) Heterotopic ossification in metastatic colorectal carcinoma: case report with morphoproteomic insights into the histogenesis. Ann. Clin. Lab. Sci. 44, 99–103 24695482

[8] Lee K. et al. (2013) Targeting of the osteoclastogenic RANKL-RANK axis prevents osteoporotic bone loss and soft tissue calcification in coxsackievirus B3-infected mice. J. Immunol. 190, 1623–1630 10.4049/jimmunol.1201479 23303667

[9] Kim S.M. et al. (2014) Osteogenetic changes in elongated styloid processes of Eagle syndrome patients. J. Craniomaxillofac. Surg. 42, 661–667 10.1016/j.jcms.2013.09.012 24161467

[10] Alam R. et al. (2013) PTEN suppresses SPARC-induced pMAPKAPK2 and inhibits SPARC-induced Ser78 HSP27 phosphorylation in glioma. Neuro Oncol. 15, 451–461 10.1093/neuonc/nos326 23382286 PMC3607267

[11] Martin E.C. et al. (2018) Trauma induced heterotopic ossification patient serum alters mitogen activated protein kinase signaling in adipose stem cells. J. Cell. Physiol. 233, 7035–7044 10.1002/jcp.26504 29377109 PMC8083017

[12] Barruet E. et al. (2018) NF-kappaB/MAPK activation underlies ACVR1-mediated inflammation in human heterotopic ossification. JCI Insight 3, pii: 122958 10.1172/jci.insight.122958 30429363 PMC6302947

[13] Atkins D. et al. (2004) Immunohistochemical detection of EGFR in paraffin-embedded tumor tissues: variation in staining intensity due to choice of fixative and storage time of tissue sections. J. Histochem. Cytochem. 52, 893–901 10.1369/jhc.3A6195.2004 15208356

[14] Ayuk S.M., Abrahamse H. and Houreld N.N. (2016) The role of photobiomodulation on gene expression of cell adhesion molecules in diabetic wounded fibroblasts *in vitro*. J. Photochem. Photobiol. B 161, 368–374 10.1016/j.jphotobiol.2016.05.027 27295416

[15] Choi Y.H. et al. (2012) Early presentation of heterotopic ossification mimicking pyomyositis - two case reports. Ann. Rehabil. Med. 36, 713–718 10.5535/arm.2012.36.5.713 23185738 PMC3503949

[16] Cairns D.M., Pignolo R.J., Uchimura T., Brennan T.A., Lindborg C.M., Xu M. et al. (2013) Somitic disruption of GNAS in chick embryos mimics progressive osseous heteroplasia. J. Clin. Invest. 123, 3624–3633 10.1172/JCI69746 23863715 PMC3726175

[17] Rosset E.M. and Bradshaw A.D. (2016) SPARC/osteonectin in mineralized tissue. Matrix Biol. 52-54, 78–87 10.1016/j.matbio.2016.02.001 26851678 PMC5327628

[18] Delany A.M. and Hankenson K.D. (2009) Thrombospondin-2 and SPARC/osteonectin are critical regulators of bone remodeling. J. Cell. Commun. Signal. 3, 227–238 10.1007/s12079-009-0076-0 19862642 PMC2778593

[19] Chiba H. and Matsuyama T. (1993) Immunohistochemical localization of bone Gla protein and osteonectin in normal human bone and cartilage tissues, and in osteosarcomas and chondrosarcomas. Nihon Seikeigeka Gakkai Zasshi 67, 463–472 8336066

[20] Sharma S. et al. (2016) Secreted protein acidic and rich in cysteine (SPARC) mediates metastatic dormancy of prostate cancer in bone. J. Biol. Chem. 291, 19351–19363 10.1074/jbc.M116.737379 27422817 PMC5016675

[21] Lanna A. et al. (2017) A sestrin-dependent Erk-Jnk-p38 MAPK activation complex inhibits immunity during aging. Nat. Immunol. 18, 354–363 10.1038/ni.3665 28114291 PMC5321575

[22] Correa R.G., Matsui T., Tergaonkar V., Rodriguez-Esteban C., Izpisua-Belmonte J.C. and Verma I.M. (2005) Zebrafish IkappaB kinase 1 negatively regulates NF-kappaB activity. Curr. Biol. 15, 1291–1295 10.1016/j.cub.2005.06.023 16051172

[23] Irelan J.T., Murphy T.J., DeJesus P.D., Teo H., Xu D., Gomez-Ferreria M.A. et al. (2007) A role for IkappaB kinase 2 in bipolar spindle assembly. Proc. Natl. Acad. Sci. U.S.A. 104, 16940–16945 10.1073/pnas.0706493104 17939994 PMC2040438

[24] McClung H.M. et al. (2012) Deletion of the SPARC acidic domain or EGF-like module reduces SPARC-induced migration and signaling through p38 MAPK/HSP27 in glioma. Carcinogenesis 33, 275–284 10.1093/carcin/bgr276 22114076 PMC3271264

[25] Wang Y. et al. (2018) Oestrogen receptor alpha regulates the odonto/osteogenic differentiation of stem cells from apical papilla via ERK and JNK MAPK pathways. Cell Prolif. 51, e12485 10.1111/cpr.12485 30069950 PMC6528913

[26] Shafiee A. et al. (2011) A comparison between osteogenic differentiation of human unrestricted somatic stem cells and mesenchymal stem cells from bone marrow and adipose tissue. Biotechnol. Lett. 33, 1257–1264 10.1007/s10529-011-0541-8 21287233

[27] Cassuto J. et al. (2018) The key role of proinflammatory cytokines, matrix proteins, RANKL/OPG and Wnt/beta-catenin in bone healing of hip arthroplasty patients. Bone 107, 66–77 10.1016/j.bone.2017.11.004 29129760

[28] Kim J.A. et al. (2016) Extracellular calcium-binding peptide-modified ceramics stimulate regeneration of calvarial bone defects. Tissue Eng. Regen. Med. 13, 57–65 10.1007/s13770-015-9066-x 30603385 PMC6170992

[29] Shen M.J. et al. (2018) Nell-1 enhances osteogenic differentiation of pre-osteoblasts on titanium surfaces via the MAPK-ERK signaling pathway. Cell. Physiol. Biochem. 50, 1522–1534 10.1159/000494651 30359975

[30] Chen D. et al. (2016) Connexin 43 promotes ossification of the posterior longitudinal ligament through activation of the ERK1/2 and p38 MAPK pathways. Cell Tissue Res. 363, 765–773 10.1007/s00441-015-2277-6 26334722

[31] Siveen K.S., Ahn K.S., Ong T.H., Shanmugam M.K., Li F., Yap W.N. et al. (2014) Y-tocotrienol inhibits angiogenesis-dependent growth of human hepatocellular carcinoma through abrogation of AKT/mTOR pathway in an orthotopic mouse model. Oncotarget 5, 1897–1911 10.18632/oncotarget.1876 24722367 PMC4039111

[32] Dan H.C., Cooper M.J., Cogswell P.C., Duncan J.A., Ting J.P. and Baldwin A.S. (2008) Akt-dependent regulation of NF-{kappa}B is controlled by mTOR and Raptor in association with IKK. Genes Dev. 22, 1490–1500 10.1101/gad.1662308 18519641 PMC2418585

[33] Tang X., Sun L., Wang G., Chen B. and Luo F. (2018) RUNX1: a regulator of NF-kB signaling in pulmonary diseases. Curr. Protein Pept. Sci. 19, 172–178 28990531 10.2174/1389203718666171009111835 PMC5876917

[34] Wang C.Q., Krishnan V., Tay L.S., Chin D.W., Koh C.P., Chooi J.Y. et al. (2014) Disruption of Runx1 and Runx3 leads to bone marrow failure and leukemia predisposition due to transcriptional and DNA repair defects. Cell Rep. 8, 767–782 10.1016/j.celrep.2014.06.046 25066130

[35] Chang H., Wang Y., Liu H., Nan X., Wong S., Peng S. et al. (2017) Mutant Runx2 regulates amelogenesis and osteogenesis through a miR-185-5p-Dlx2 axis. Cell Death Dis. 8, 3221 10.1038/s41419-017-0078-4 29242628 PMC5870583

[36] Wang L., Lee W., Cui Y.R., Ahn G. and Jeon Y.J. (2019) Protective effect of green tea catechin against urban fine dust particle-induced skin aging by regulation of NF-kappaB, AP-1, and MAPKs signaling pathways. Environ. Pollut. 252, 1318–1324 10.1016/j.envpol.2019.06.029 31252129

[37] Wang Y. et al. (2014) Mineral trioxide aggregate upregulates odonto/osteogenic capacity of bone marrow stromal cells from craniofacial bones via JNK and ERK MAPK signalling pathways. Cell Prolif. 47, 241–248 10.1111/cpr.12099 24635197 PMC6496412

